# Application of the PET ligand [^11^C]ORM-13070 to examine receptor occupancy by the α_2C_-adrenoceptor antagonist ORM-12741: translational validation of target engagement in rat and human brain

**DOI:** 10.1186/s13550-020-00741-y

**Published:** 2020-12-09

**Authors:** Mohammed Shahid, Juha O. Rinne, Mika Scheinin, Jere Virta, Päivi Marjamäki, Olof Solin, Eveliina Arponen, Jukka Sallinen, Katja Kuokkanen, Juha Rouru

**Affiliations:** 1 Orion Corporation, Orion Pharma, Research and Development, Tengströminkatu 8, 20380 Espoo, Finland; 2 Turku PET Centre, University of Turku and Turku University Hospital, Turku, Finland; 3 Division of Clinical Neurosciences, Turku University Hospital, Turku, Finland; 4 CRST, Turku, Finland; 5 Institute of Biomedicine, University of Turku, Turku, Finland; 6 Unit of Clinical Pharmacology, Turku University Hospital, Turku, Finland; 7 MediCity Research Laboratory, University of Turku, Turku, Finland; 8 Department of Chemistry, University of Turku, Turku, Finland; 9 Accelerator Laboratory, Åbo Akademi University, Turku, Finland; 10 Orion Corporation, Orion Pharma, Research and Development, Nottingham, UK

**Keywords:** Brain α_2C_-adrenoceptors, Receptor occupancy, ORM-12741, [^11^C]ORM-13070 PET, α_2C_-Adrenoceptor antagonists

## Abstract

**Background:**

Availability of the α_2C_-adrenoceptor (α_2C_-AR) positron emission tomography (PET) tracer, [^11^C]ORM-13070, and the α_2C_-AR antagonist ORM-12741 allows probing of the roles of this G-protein coupled receptor subtype in brain function, both in healthy humans and in patients with various brain disorders. This translational study employed [^11^C]ORM-13070 autoradiography and PET to determine α_2C_-AR occupancy by ORM-12741 in rat and human brain, respectively.

**Results:**

ORM-12741 has high affinity (*K*_i_: 0.08 nM) and potent antagonist activity (*K*_b_: 0.04 nM) as well as selectivity (*K*_i_ estimates for the human α_2A_-AR and α_2B_-AR were 8.3 nM and 0.8 nM, respectively) for the human α_2C_-AR subtype. [^11^C]ORM-13070 had highest uptake in the basal ganglia of rat and human brain. Pretreatment with ORM-12741 inhibited [^11^C]ORM-13070 binding in rat striatum in a time- and dose-dependent manner at 10 and 50 µg/kg (s.c.) with an EC_50_ estimate of 1.42 ng/mL in rat plasma, corresponding to protein-free drug concentration of 0.23 nM. In the living human brain, time- and dose-related α_2C_-AR occupancy was detected with EC_50_ estimates of 24 ng/mL and 31 ng/mL for the caudate nucleus and putamen, respectively, corresponding to protein-free concentrations in plasma of 0.07 nM and 0.1 nM. Modelling-based maximum α_2C_-AR occupancy estimates were 63% and 52% in the caudate nucleus and the putamen, respectively.

**Conclusions:**

ORM-12741 is a selective α_2C_-AR antagonist which penetrates the rat and human brain to occupy α_2C_-ARs in a manner consistent with its receptor pharmacology.

*Trial* *registration* *number* *and* *date* *of* *registration*: ClinicalTrial.cov NCT00829907. Registered 11 December 2008. https://clinicaltrials.gov/.

## Background

An inhibitory G-protein coupled receptor of the neurotransmitter noradrenaline (NA), the α_2C_-adrenoceptor (α_2C_-AR) subtype, has attracted considerable interest as a therapeutic target to treat CNS disorders [1]. The α_2C_-AR may be involved in mediation of the fine-tuning effects of NA on central neurotransmission, particularly during stressful conditions. Results obtained with gene-targeted (knock-out) mice indicate that manipulation of α_2A_-AR and α_2C_-AR activation yields differential behavioural effects in nonclinical tests that are commonly used for assessing antidepressant, antipsychotic or pro-cognitive properties of drugs [1]. This has led to the proposition that selective α_2C_-AR antagonism might be a promising approach for the treatment of neuropsychiatric symptoms, potentially across a wide range of CNS disorders, with an improved therapeutic profile compared to non-selective α_2_-AR antagonists [1].

The availability of the novel α_2C_-AR antagonist ORM-12741 and the α_2C_-AR positron emission tomography (PET) tracer [^11^C]ORM-13070 now provides novel opportunities to investigate the roles and possible therapeutic utility of α_2C_-AR modulation in CNS disorders [1]. Of all known α_2C_-AR antagonists, ORM-12741 is the most advanced molecule in terms of data on human exposure; it is rapidly absorbed after oral dosing and has shown acceptable tolerability [2]. Direct evidence supporting drug target engagement is a key element for establishing confidence in proof of concept evaluation in nonclinical and human studies [3]. [^11^C]ORM-13070 as a PET tracer has provided a valuable probe for specifically investigating α_2C_-AR subtype functions as well as brain receptor occupancy in experimental animals and humans. Its application as a PET tracer has been validated and established in several studies, with acceptable test–retest reproducibility [4–7]. Furthermore, additional work has shown that [^11^C]ORM-13070 binding is sensitive to changes in extracellular NA concentrations in the human brain, provoked by physiological or pharmacological interventions, indicating that it may be a valuable tracer for the investigation of alterations in noradrenergic tone [8, 9].

## Methods

The current translational investigation was aimed at demonstrating target receptor engagement for ORM-12741 as well as establishing the utility of [^11^C]ORM-13070 as a suitable PET tracer for assessment of α_2C_-AR occupancy in rat and human brain.

### α_2_-AR subtype binding and antagonist characteristics in vitro

Receptor binding assays were performed at Cerep (Celle l’Evescault, France) according to their standard procedures, using stably transfected cell lines. Inhibition constants (*K*_i_) were calculated using the Cheng–Prusoff equation [10]. CHO cells transfected to express human α_2_-AR subtypes were used to determine antagonist properties of ORM-12741 in a calcium ion based fluorescent assay as described previously [11]. Adrenaline and noradrenaline were used as agonists, and changes in intracellular calcium were monitored with a FLEXstation bench top scanning fluorometer equipped with an integrated fluid transfer workstation (Molecular Devices, San Jose, CA, USA) and SOFTmax PRO version 3.2 software. ORM-12741 (Orion Pharma, Espoo, Finland; 10^–2^ M) was dissolved in DMSO and subsequently diluted in Probenecid Ringer buffer.

### Radiosynthesis of [^11^C]ORM-13070

[^11^C]ORM-13070 was synthesized at Turku PET Centre Radiopharmaceutical Laboratory, Turku, Finland as described previously [4] and was dissolved in a mixture of propylene glycol/ethanol/0.1 M phosphate buffer (7/3/45, v/v/v), pH 7.4 [4].

### Rat brain ex vivo autoradiography

An ex-vivo autoradiography method, as described in [4], based on specific displacement of [^11^C]ORM-13070 binding in the caudate-putamen nucleus, was used to determine α_2C_-AR occupancy in rat brain. Male Sprague–Dawley rats (*n* = 4–6/group) were treated with vehicle (PEG 300/5% glucose) or ORM-12741 (dissolved in PEG 300 and diluted with 5% glucose solution; 2, 10, 50 or 1000 µg/kg, s.c.) 10 min before injection of [^11^C]ORM-13070 (38–81 MBq) into the tail vein. Body temperature was kept stabile using a heating mattress. At 10 or 30 min after [^11^C]ORM-13070 administration, the rats were stunned by CO_2_ asphyxiation and terminal blood samples were taken for determination of plasma levels of ORM-12741. Brains were frozen by immersion in isopentane chilled on CO_2_ ice. Cryosections (40 µm) of the brain were prepared and regions of interest (caudate-putamen/cerebellum) were analysed by autoradiography as described previously by the Aida 2D densitometry program [4]. Occupancy calculations were done similarly to the clinical study described below but instead of the baseline, average values in the vehicle group were used.

Animal care complied with the guidelines of the International Council of Laboratory Animal Science. The Animal Experiment Board of the Province of Southern Finland approved the methodologies used in this study.

### α_2C_-AR occupancy in human brain in vivo

The clinical trial was an open label, single dose, uncontrolled study performed at a single centre. The primary objective of the study was to determine the extent of brain α_2C_-AR occupancy after different single oral doses of ORM-12741 and to describe the relationship of α_2C_-AR occupancy as a function of ORM-12741 dose and drug concentration in plasma. The study design involved dose ranging with adaptive selection of doses and assessment time points. The study protocol was approved by the Ethics Committee of the Hospital District of Southwest Finland and the Finnish Medicines Agency (EudraCT 2008-004929-42), and the trial was registered in the ClinicalTrials.gov database (NCT00829907).

Healthy male volunteers were enrolled after informed consent. Concomitant medications that could have affected the outcome of the study were prohibited within 2 weeks prior to the first PET scan or less than 5 times the elimination half-life of the medication. The use of nicotine containing products were forbidden during the stay at the study centre. Drug abuse and alcohol breath test were performed prior to PET scans and had to be negative. Each subject had three visits: a screening visit, a treatment visit and an end-of-study safety visit. A brain MRI scan was obtained for an individual anatomical reference map. All included subjects had a baseline PET scan ([^11^C]ORM-13070 alone) and 1–3 scans at set time points after different doses of ORM-12741 (Table 1).

**Table 1:** Numbers of subjects in each dose group and PET scan time points after oral dosing with the α_2C_-adrenoceptor antagonist ORM-12741

Dose (mg)	No. of subjects scanned at baseline	No. of subjects scanned at 1 h	No. of subjects scanned at 3.5 h	No. of subjects scanned at 6 h	No. of subjects scanned at 6.5 h	No. of subjects scanned at 12 h
0.3	2	1	1	1		1
1	3^a^	1	1	1		1
10	5	3	3	2		2
30	5	3	3	2		2
60	4	4	4		4	

^a^One subject discontinued after the baseline scan

Soft gelatin capsules containing ORM-12741 (0.1 mg, 1 mg and 10 mg) were produced by Orion Pharma (Espoo, Finland), and each study subject received a single oral dose (0.3, 1, 10, 30 or 60 mg) (Table 1).

Each [^11^C]ORM-13070 dose (target radioactivity 550 MBq; < 10 μg of ORM-13070) was given as a rapid intravenous bolus injection (1–10 mL) at the start of the PET scan. PET imaging was performed as described previously [7]. In brief a high-resolution research tomograph (HRRT; Siemens Medical Solutions, Knoxville, TN) with the subject’s head fixed in a head holder with an individually prepared thermoplasticmask was used. In addition, head movements were recorded with an infrared camera (Vicra®; Northern Digital Inc., Waterloo, ON, Canada). Slices of approximately 1.22 mm thickness covered the whole brain (axial field of view 25.3 cm). The camera was used in 3-D mode with scatter correction. The HRRT achieved transaxial and axial spatial resolution (full-width at half-maximum) of 2.5 mm. Before each PET scan, a transmission scan was done for attenuation correction with a ^137^Cs rotating point source. Regions-of-interest (ROIs) were manually drawn on the co-registered MRI scans using Imadeus software (version 1.1, Forima, Turku, Finland), checked to match the summated PET images and then transferred onto the dynamic PET image, from which regional time-activity curves were obtained for the following selected regions of the left and right brain hemispheres: caudate nucleus, cerebellar cortex and putamen as described previously [7].

Tracer uptake in the ROIs was described with areas under the curves (AUC) in the scan time window of 5–30 min after tracer injection. As the cerebellum has been reported to be devoid of α_2C_-ARs, it was used as a reference region for correction of non-specific uptake. A binding parameter (BiP) was calculated for each ROI as the ratio of specific binding (AUC_region _− AUC_cerebellar cortex_) and the AUC in the cerebellar cortex. Receptor occupancy by [^11^C]ORM-13070 was negligible at the employed tracer doses (< 10 μg) [7]. Receptor occupancy in the target regions was calculated according to the equation:

$$\% \;{\text{Receptor}}\;{\text{occupancy}} = \left( {1 - \frac{{{\text{BiPdrug}}}}{{{\text{BiPbaseline}}}}} \right) \times 100\%$$

where BiP_baseline_ = pre-drug baseline BiP value, BiP_drug_ = BiP value following ORM-12741.

Left- and right-side receptor occupancy estimates were averaged to a single value for each ROI.

Liquid chromatography-tandem mass spectrometry was used for the determination of concentrations of ORM-12741 in plasma extracted from venous blood samples that were collected before and 10 min, 40 min, 60 min 90 min, 2 h, 3.5 h, 4 h, 6 h, 6,5 h,12 h, 12.5 h and 24 h after ORM-12741 dosing. Plasma PK variables (*C*_max_: peak concentration, *t*_max_: time to peak concentration, AUC_t_: area under the drug plasma concentration–time curve from time zero to the last observed concentration, AUC_∞_: area under the drug plasma concentration–time curve from time zero to infinity, *t*_1/2_: terminal half-life) for ORM-12741 were calculated by non-compartmental analysis using the WinNonlin® Professional software package version 5.0.1 (Pharsight Corporation, Mountain View, CA, USA). The actual time points for blood sampling were used in the PK calculations.

Nonlinear regression analysis was used to evaluate the relationships between ORM-12741 plasma levels and receptor occupancy (Sigmoid *E*_max_ model):

$${\text{Occupancy}} = \frac{{E_{\max } *C^{h} }}{{{\text{EC}}_{50}^{h} + C^{h} }}$$

where *E*_max_ is a maximum receptor occupancy estimate, EC_50_ is a half maximal effective concentration estimate and h is a slope factor. Temporal occupancy patterns were estimated with a regression model. Statistical analyses were performed with SAS® for Windows (SAS Institute Inc., Cary, NC, USA) on observed cases only.

The safety of the subjects was evaluated by recording of adverse events (AEs), supine heart rate and blood pressure, 12-lead electrocardiogram, laboratory safety assessments and physical examination findings.

## Results

### In vitro human receptor pharmacology

ORM-12741 displayed high affinity and potent antagonist activity as well as selectivity for the human α_2C_-AR. The K_i_ estimates for the human α_2A_-AR, α_2B_-AR and α_2C_-AR were 8.3, 0.8 and 0.08 nM, respectively. In functional assays, ORM-12741 inhibited adrenaline-induced elevations of intracellular calcium mediated by human α_2A_-AR, α_2B_-AR and α_2C_-AR with equilibrium dissociation constant (*K*_b_) estimates of 55, 1.4 and 0.04 nM, respectively. Similar antagonist potency estimates were obtained when noradrenaline was used as the agonist, with K_b_ estimates of 41, 5.6 and 0.01 nM for α_2A_-AR, α_2B_-AR and α_2C_-AR, respectively.

### Rat brain ex vivo autoradiography

**Fig. 1:**
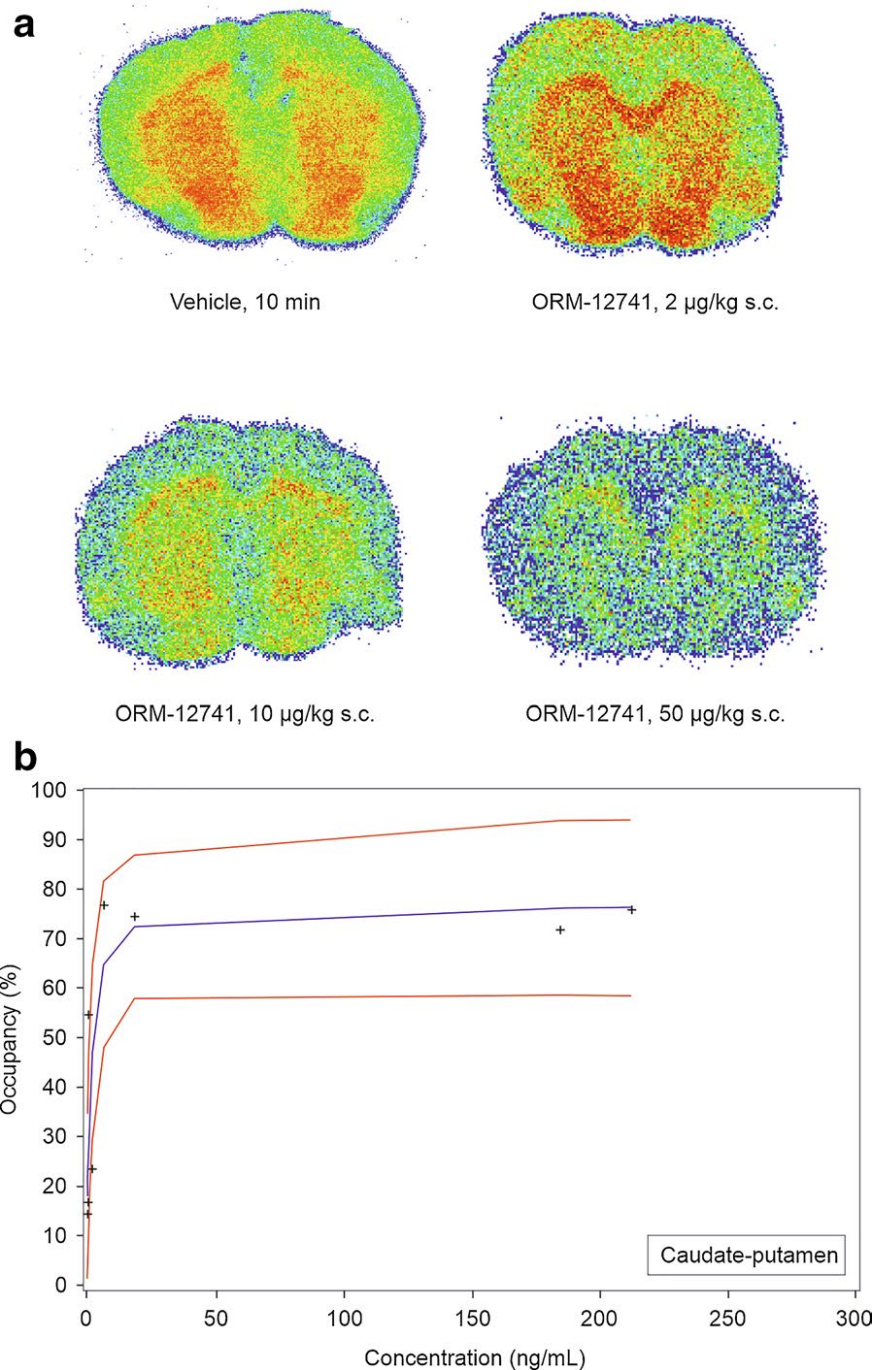
**a** Representative 40 µm coronal brain sections from rats pre-treated with vehicle or ORM-12741 (2, 10, 50 µg/kg subcutaneously) at 10 min post [^11^C]ORM-13070 intravenous injection. Sections are from the level of the caudate-putamen, olfactory tubercle and frontal cortex. The colour pseudo-autoradiograms show the regional distribution of the [^11^C]-label, red highest, blue lowest uptake. **b** The relationship between ORM-12741 (10–1000 µg/kg s.c.) plasma levels and α_2C_-adrenoceptor occupancy in rat striatum. The blue line represents a binding hyperbole derived from nonlinear regression analysis with a sigmoidal maximum possible effect model. The red lines represent 95% confidence intervals

Ex vivo brain autoradiography with [^11^C]ORM-13070 produced the strongest signals in the striatum, with less tracer uptake in other brain regions, such as the hippocampus and frontal cortex (Fig. 1a). The signal was most intense 10 min after the injection of [^11^C]ORM-13070 and had dissipated by 30 min. Pretreatment with ORM-12741 inhibited the uptake of [^11^C]ORM-13070 in a time- and dose-dependent manner, indicating engagement of α_2C_-ARs, with clear inhibitory effects after doses ranging from 10 to 1000 µg/kg s.c. (Fig. 1b). Based on a tentative analysis of the limited available dataset, a half maximal effective concentration (EC_50_) value of 1.42 ng/mL (95% confidence interval (CI): 0.55–3.65 ng/mL) was determined for concentrations of ORM-12741 in plasma, corresponding to an unbound fraction of 0.074 ng/mL (0.23 nM) and a maximum receptor occupancy estimate (*E*_max_) of 76% (CI: 60–98%).

### Human brain α_2C_-AR occupancy in vivo

A total of 26 male subjects were screened and 19 were included in the study (mean age 24.5 years; range 18–40 years, mean body mass index 23.3 kg/m^2^; range 20–28 kg/m^2^). One subject in the 1 mg dose group discontinued the study due to personal reasons after his baseline PET scan and did not receive ORM-12741. Eighteen subjects completed the study and were administered single oral doses of ORM-12741 (Table 1). The median radioactive dose of [^11^C]ORM-13070 was 499 (range 302–558) MBq for baseline PET scans and 489 (range 209–523) MBq for scans after ORM-12741 administration. The median injected mass of [^11^C]ORM-13070 was 0.4 (range 0.1–2.9) μg for baseline PET scans and 0.4 (range 0.1–1.9) μg for PET scans after ORM-12741 administration.

**Fig. 2:**
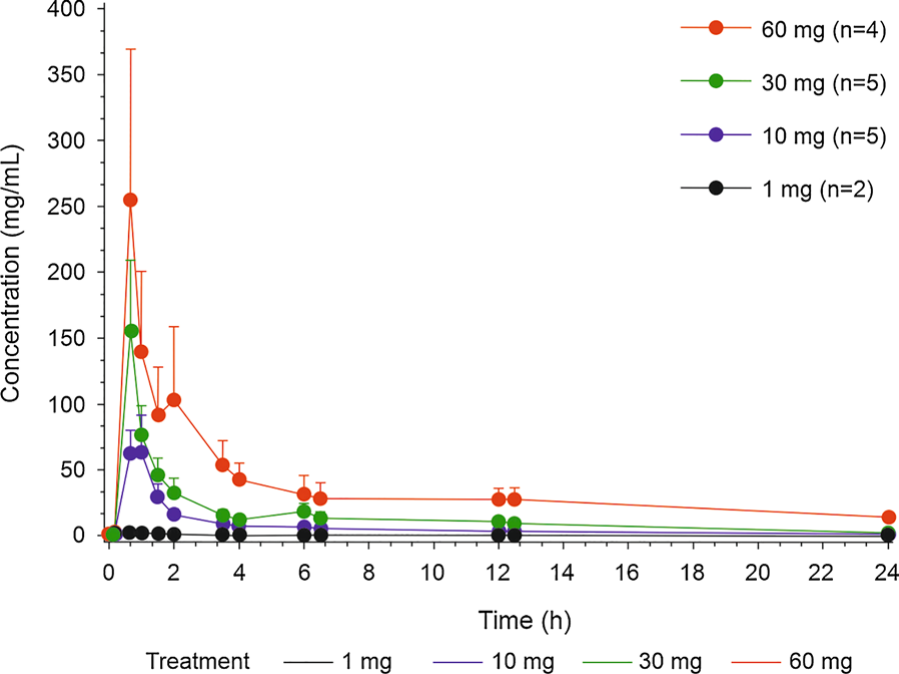
Pharmacokinetic profile of ORM-12741 showing mean (± standard error of mean) plasma concentrations following oral administration of four single doses to fasted healthy male volunteers

Following oral administration, plasma levels of ORM-12741 increased rapidly and peaked between 0.7 and 1.1 h, with median t_max_ ranging from 0.7 to 0.9 h for the different doses (Fig. 2 and Additional file 1: Table S1).

Regional brain α_2C_-AR occupancy (in the caudate nucleus and putamen), as measured by [^11^C]ORM-13070 PET analysis, after single oral doses of ORM-12741 is summarized in Additional file 1: Table S2.

In the baseline scans, the largest BiP estimates were seen in the caudate nucleus and putamen. The α_2C_-AR occupancy by ORM-12741 was also most evident in these ROIs. Since good test–retest reproducibility for [^11^C]ORM-13070 uptake has been previously demonstrated in these brain regions [6], occupancy results from these ROIs are presented in more detail. Individual α_2C_-AR occupancy results by time point in the caudate nucleus and putamen and corresponding ORM-12741 concentrations in plasma are presented in Additional file 1: Table S3.

**Fig. 3:**
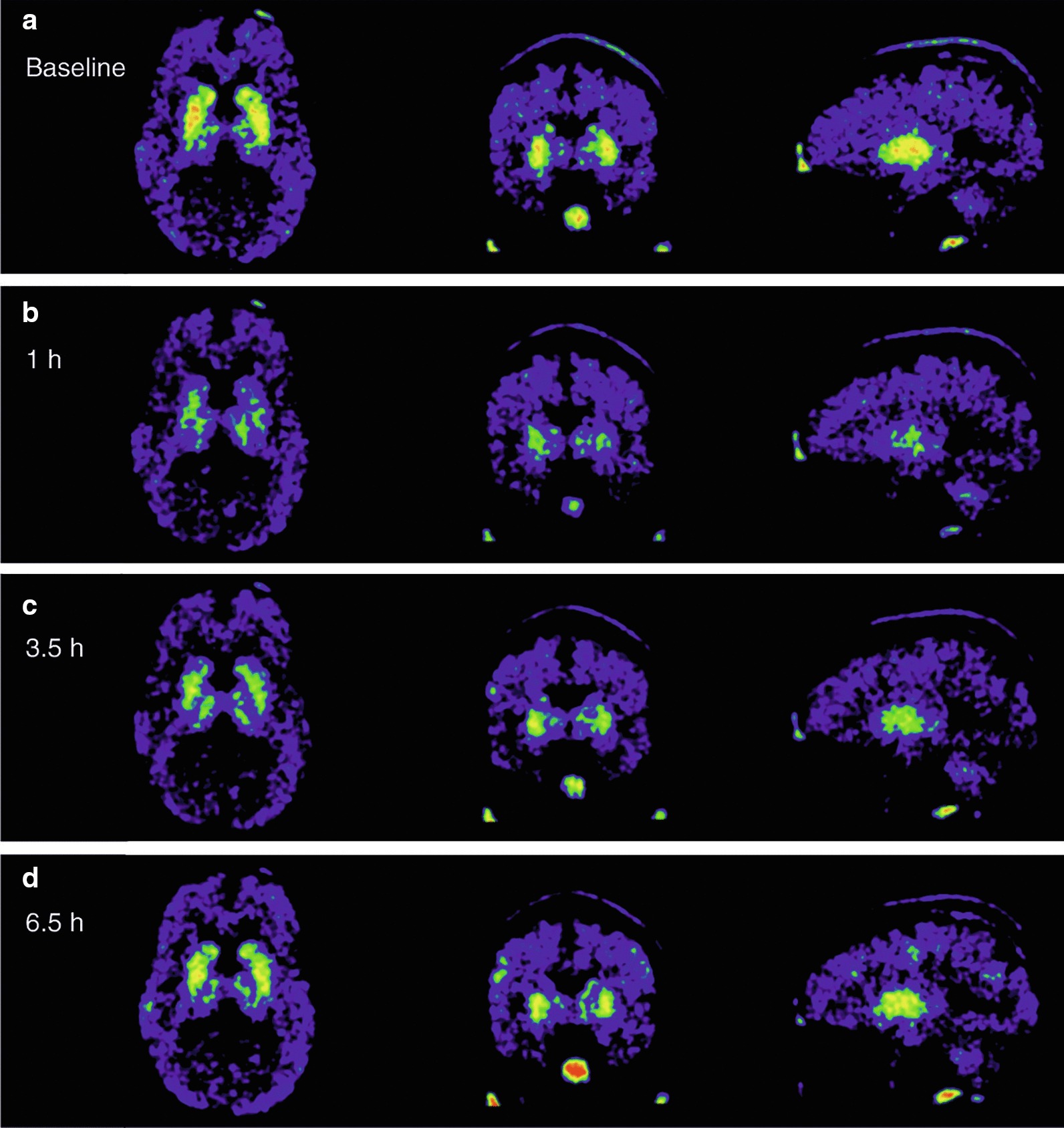
A representative set of human brain PET SUV images from one healthy male volunteer at baseline and 1 h, 3.5 h and 6.5 h after 60 mg of ORM-12741

**Fig. 4:**
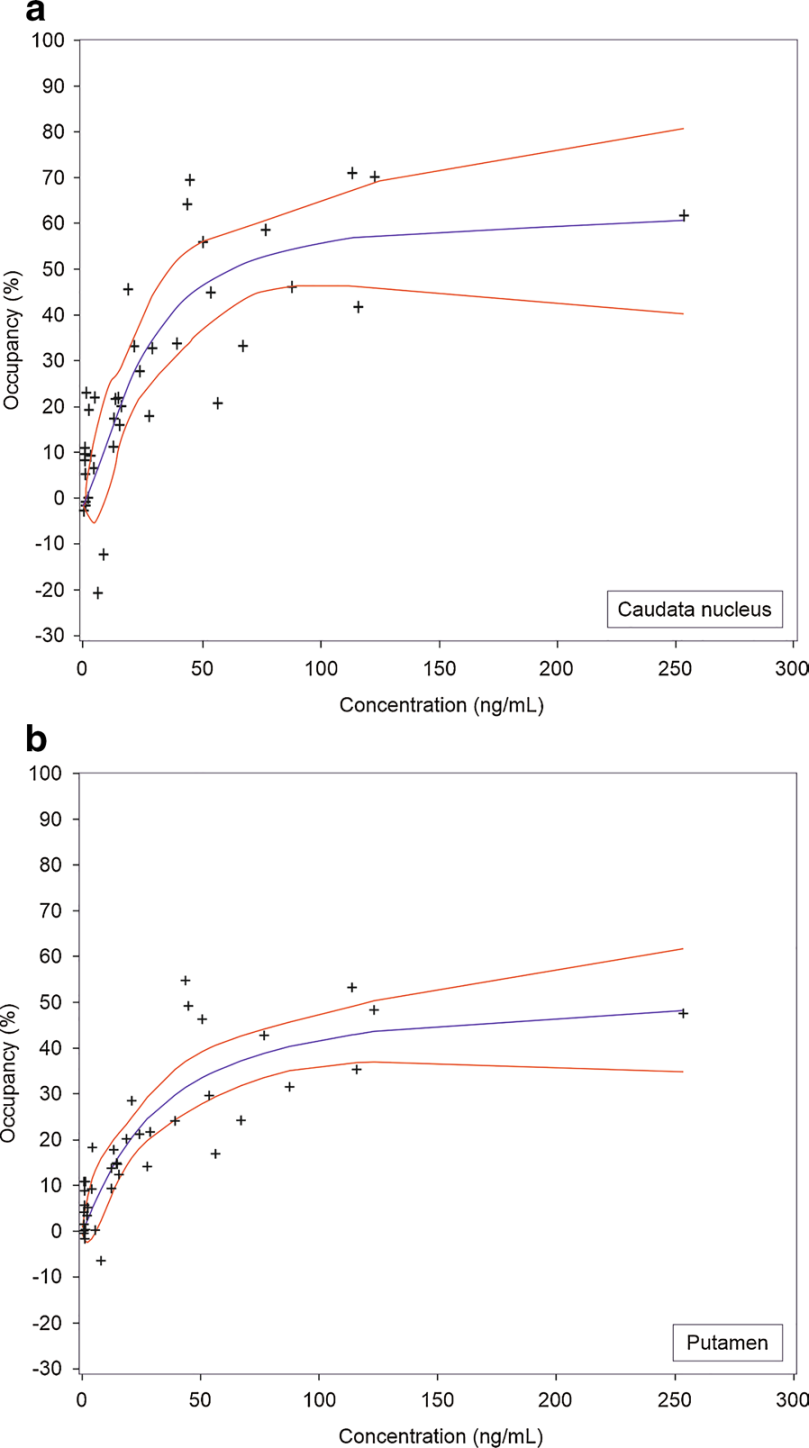
The relationship between ORM-12741 plasma levels and α_2C_-adrenoceptor occupancy in human **a** caudate nucleus and **b** in putamen. The blue line represents binding hyperboles derived from nonlinear regression analysis with a sigmoidal maximum possible effect model. The red lines represent 95% confidence intervals. Concentrations of ORM-12741 in plasma represent mean values of samples collected at the start and end of the PET scan. Each data point represents an individual occupancy value at one time point

In the caudate nucleus and putamen, ORM-12741 produced dose-related increases in α_2C_-AR occupancy up to the 30 mg dose, with little or no effect seen at the 0.3 and 1 mg dose levels. After 10, 30 or 60 mg of ORM-12741, significant receptor occupancy was observed, peaking at one hour after dosing with occupancy estimates up to 42%, 70% and 71%, respectively, in the caudate nucleus. Figure 3 shows a representative set of PET images from one subject who received 60 mg of ORM-12741. In the putamen, the occupancy estimates were generally somewhat lower than in the caudate nucleus (Fig. 4a, b).

Based on sigmoidal maximum possible effect (*E*_max_) modelling, the *E*_max_ estimates (with 95% CIs) were 63 (CI: 39–100) % as calculated from the caudate nucleus data (Fig. 4a) and 52 (CI: 31–89) % as calculated from the putamen data (Fig. 4b). The half-maximal effective concentration (EC_50_) estimates for ORM-12741 in plasma (with 95% CIs) were 24 (10–58) ng/mL and 31 (9.8–97) ng/mL, corresponding to an unbound fraction of 0.024 ng/mL (0.07 nM) and 0.031 ng/mL (0.1 nM) for the caudate nucleus (Fig. 4a) and putamen (Fig. 4b), respectively. The corresponding EC_90_ estimates (concentrations that produce 90% of the maximum effect) were 105 ng/mL and 209 ng/mL for the caudate nucleus and putamen, respectively.

Both ORM-12741 and [^11^C]ORM-13070 were well tolerated, with no serious AEs. Eleven mild AEs were reported in 8 subjects. Headache was the most common AE (4 events in 4 subjects) All AEs occurring after ORM-12741 administration are presented in Supplemental Material Table S4.

## Discussion

The current translational investigation provides evidence supporting ORM-12741 as a selective, high-affinity antagonist of α_2C_-ARs with sufficient penetration of the blood–brain barrier to occupy α_2C_-ARs in the human brain, confirming its primary mode of action.

Receptor binding analysis demonstrated that ORM-12741 has high affinity for the cloned human α_2C_-AR (*K*_i_: 0.08 nM) and lower affinity for the α_2A_-AR (*K*_i_: 8.3 nM) and α_2B_-AR (*K*_i_: 0.8 nM) subtypes, i.e. approximately 100- and 10-fold receptor subtype selectivity. ORM-12741 also antagonized intracellular calcium responses mediated by cloned human α_2C_-AR activated with adrenaline (*K*_b_: 0.04 nM) or noradrenaline (*K*_b_: 0.01 nM) with potency estimates consistent with its binding affinity. Its relative α_2_-AR subtype selectivity was somewhat higher in the functional assay compared to the receptor binding assay, with 4100- and 560-fold higher potency at the α_2C_-AR compared to α_2A_-AR (*K*_b_: 41 nM) and α_2B_-AR (*K*_b_: 5.6 nM). In a general selectivity screen with 126 additional receptors and drug binding sites (GPCRs, ion channels, transporters, enzymes), binding of ORM-12741 to the α_1A_-AR (*K*_i_ estimate, 46 nM) was most notable, but this represented an approximately 575-fold affinity ratio when compared with α_2C_-AR. ORM-12741 had much lower affinity (α_2C_-AR selectivity at least > 2000 fold) against all other targets tested (unpublished data, Orion Pharma). Overall, these results confirm that ORM-12741 is a selective, high-potency antagonist of human α_2C_-ARs. Since the α_2A_-AR is the most prevalent and widely distributed α_2_-AR subtype in humans, high selectivity over this target should reduce the potential for peripheral (e.g. cardiovascular) or central (e.g. anxiety) side-effects that are commonly observed with subtype non-selective α_2_-AR antagonists [12, 13].

[^11^C]ORM-13070 has previously been validated as a selective PET ligand for assessing α_2C_-AR expression and occupancy in rat [4] and human brain [7]. The current results extend and support these previous findings, confirming that [^11^C]ORM-13070 shows similar regional distribution patterns in rat and human brain, with the most intense signal in the striatum. In an ex vivo autoradiography experiment pretreatment of rats with ORM-12741 inhibited [^11^C]ORM-13070 binding in a dose- and exposure-related manner with significant effects at 10 µg/kg (s.c.). This dose was associated with a *C*_max_ in rat plasma of 3–6 nM, and a protein-unbound free drug concentration of 0.015–0.03 nM (free fraction 5% in rat plasma), which is in line with the affinity of ORM-12741 for α_2C_-AR in vitro. Furthermore, similar exposure levels have been associated with the pharmacodynamic effects of ORM-12741 seen in the rat forced swim test (FST) and the phencyclidine-induced prepulse inhibition (PPI) model at doses of ≥ 16 µg/kg (s.c.) and ≥ 10 µg/kg (s.c.), respectively [14]. Consistently, gene-targeted α_2C_-AR knock-out mice have shown reduced immobility in the FST [15, 16]. In addition, other α_2C_-AR antagonists have shown similar effects in the FST and PPI models [1, 11, 17]. Collectively, the accumulated in vitro and in vivo receptor-level evidence, together with the phenotypic pharmacodynamic signals observed in the FST and PPI models, formed the basis for this translational study in human subjects to validate the engagement of brain α_2C_-ARs by ORM-12741.

The current PET study further confirmed the previously reported [^11^C]ORM-13070 uptake and distribution pattern in the human brain, with the strongest binding signal being observed in the caudate nucleus and putamen [6, 7]. This is also in line with the known distribution of α2C-adrenoceptors in post-mortem human brain samples, i.e. high in the caudate nucleus and putamen, low in cortex and neglible in cerebellum [18]. Given the very small mass (average, 0.4 µg) of ORM-13070 delivered with the target radioactivity, the PET tracer was unlikely to compromise receptor availability for the occupancy analysis. These features of [^11^C]ORM-13070 together with acceptable PK properties and good test–retest reliability make it a feasible tracer for PET-based receptor occupancy analysis. The results obtained with ORM-12741 in the present investigation provide further support for this notion, for the first time employing a subtype-selective α_2C_-AR antagonist. Dosing with ORM-12741 decreased the specific binding of [^11^C]ORM-13070 in the caudate nucleus and putamen in a time- and exposure-dependent manner, indicating occupancy of α_2C_-ARs.

Significant α_2C_-AR occupancy was detectable in the human brain after ≥ 10 mg oral doses of ORM-12741. The peak receptor occupancy and the time course of occupancy were in agreement with drug concentrations in plasma, in terms of e.g. *C*_max_ and *t*_max_. The observed mean *C*_max_ of ORM-12741 in plasma after 10 mg doses was 62.6 ng/mL, corresponding with a protein-unbound free drug concentration of 0.2 nM, which is close to its in vitro *K*_i_ estimate (0.08 nM) for the α_2C_-AR. Based on the measured plasma concentrations after 30 mg and 60 mg doses of ORM-12741, and taking into account an approximately 0.1% free fraction in human plasma, these doses yielded approximately 0.4 nM and 0.5 nM free concentrations of ORM-12741 in plasma, respectively. These estimates are broadly consistent with the results obtained in vitro with cloned human α_2C_-AR, indicating that 1 nM ORM-12741 produces 93% inhibition of (–)adrenaline binding. Furthermore, 1 nM ORM-12741 did not affect (–)adrenaline binding to the α_2A_-AR, suggesting that the doses used in the current PET study are likely to reflect selective antagonism of α_2C_-ARs. The low doses of 0.3 and 1 mg of ORM-12741 resulted in average *C*_max_ of 1.2 ng/mL and 2.7 ng/mL, respectively, which provided free drug concentrations (4–9 pM) well below its α_2C_-AR K_i_, explaining the lack of displacement of [^11^C]ORM-13070 after these doses. At the 10–60 mg dose levels, the basal ganglia occupancy estimates reached their maximum at about 1 h after dosing and then declined at the 6 and 12 h time points towards minimal residual occupancy. The maximum occupancy was increased in a dose-related fashion up to the 30 mg dose level (about 70% in the caudate nucleus), but increasing the dose further to 60 mg did not increase occupancy at 1 h. Still, the occupancy estimates at 3.5 h were somewhat higher after 60 mg than after 30 mg.

The relationship of α_2C_-AR occupancy in the caudate nucleus and putamen with plasma ORM-12741 concentrations was best described by a sigmoidal *E*_max_ model, in concordance with classical receptor binding to a single population of receptors. The analysis of the relationship of α_2C_-AR occupancy with ORM-12741 concentrations in plasma was limited by the paucity of PET scanning data at higher plasma concentrations of ORM-12741 than 125 ng/mL. Thus, further increases in regional brain α_2C_-AR occupancy with increasing concentrations of ORM-12741 in plasma can therefore not be excluded. Therefore, the *E*_max_, EC_90_ and EC_50_ estimates should be viewed as preliminary estimates. However, the maximum occupancy estimates achieved in the present study were in the same range as previous results where [^11^C]ORM-13070 occupancy was measured in healthy human subjects after administration of the subtype-nonselective α_2_-AR antagonist atipamezole [7], and are also in line with the rat ex vivo autoradiography data. Issues to be considered in this context include the relative receptor binding specificity of the two competing α_2C_-AR ligands, the tracer and the test drug ORM-12741, and the contribution of a putative radioactive tracer metabolite that may have interfered with the occupancy estimation. The results of an earlier validation study of the α_2C_-AR PET tracer [^11^C]ORM-13070 [7], supported by nonclinical observations [4], indicated that a radioactive tracer metabolite may enter the brain but appears to exhibit no specific binding to α_2C_-ARs. Therefore, a negative bias may be present in the BiP estimates. Longer scan times would be expected to lead to even greater bias in BiP due to the accumulation of the metabolite in the brain. It is also noteworthy that it was not possible to include time as a factor into the model. Thus, it seems plausible that the occupancy estimates follow plasma ORM-12741 concentrations relatively closely.

At the time of the human PET study a validated simplified activity ratio method was used for receptor occupancy determination. This was based on extensive validation in a series of earlier studies with the same PET ligand [6–9]. However, it is important to emphasize that this may be a significant limitation as the activity ratio method used does not allow to take into account any medication related changes in blood flow. Using the reference tissue approach for determination of binding potential may have been more effective in this respect. In addition, due to unfortunate circumstances MRI images were not available for additional analyses any more.

Species differences in the binding of ORM-12741 to plasma proteins are likely to explain the difference in total exposure levels required for effects in humans and rats; the free fraction is ~ 50-fold higher in rat plasma compared to human plasma. Overall, the PET study results provide direct evidence to support the primary mode of action of ORM-12741, involving α_2C_-AR occupancy in the human brain.

## Conclusion

The current translational investigation provides evidence to support ORM-12741 as a novel, selective α_2C_-AR antagonist with target engagement demonstrated both in rat and human brain in a manner consistent with its receptor pharmacology. The results thus help to confirm its primary mechanism of action, involving selective occupancy of α_2C_-AR in the basal ganglia of the rat and human brain, and also provides valuable insight for dose selection in patient trials. Indeed, the present results were already used to help to decide a dosing scheme of ORM-12741 associated with meaningful α_2C_-AR occupancy, to be used in a Phase 2 clinical drug trial in patients with Alzheimer´s disease [2].

## Supplementary information

**Additional file 1:** Tables S1–S4.

## Data Availability

The datasets generated and analysed during the current study are not publicly available due to intellectual property reason, but are available from the corresponding author on reasonable request.

## Abbreviations

NA: Noradrenaline
α_2C_-AR: α_2C_-Adrenoceptor
K_i_: Inhibition constant
ROI: Region-of-interest
AUC: Area under the curve
BiP: Binding parameter
K_b_: Equilibrium dissociation constant
EC_50_: A half maximal effective concentration
E_max_: A maximum receptor occupancy estimate
E_max_: Maximum possible effect
FST: Forced swim test
PPI: Prepulse inhibition
