# Supplementary material for: Application of the PET ligand [11C]ORM-13070 to examine receptor occupancy by the α2C-adrenoceptor antagonist ORM-12741: translational validation of target engagement in rat and human brain
Source: EJNMMI Res. 2020 Dec 9;10:152. doi: 10.1186/s13550-020-00741-y (PMC7726058; doi:10.1186/s13550-020-00741-y)
Supplement: Supplementary file 1 — Additional file 1: Tables S1–S4. [file 13550_2020_741_MOESM1_ESM.docx]

**Additional file 1**

**Application of the PET ligand [^11^C]ORM-13070** **to examine receptor occupancy by the α_2C_-adrenoceptor antagonist ORM-12741: translational validation of target engagement in rat and human brain**

Mohammed Shahid^1^, Juha O. Rinne^2,3^, Mika Scheinin^4,5,6^, Jere Virta^2,3^, Päivi Marjamäki^2,7^, Olof Solin^2,8,9^, Eveliina Arponen^2^, Jukka Sallinen^1^, Katja Kuokkanen^1^, Juha Rouru^1^

Affiliations:

^1^Orion Corporation, Orion Pharma, Research and Development, Espoo, Finland

^2^Turku PET Centre, University of Turku and Turku University Hospital, Turku, Finland

^3^Division of Clinical Neurosciences, Turku University Hospital, Turku, Finland

^4^CRST, Turku, Finland

^5^Institute of Biomedicine, University of Turku, Finland

^6^Unit of Clinical Pharmacology, Turku University Hospital, Turku, Finland

^7^MediCity Research Laboratory, University of Turku, Turku, Finland

^8^Department of Chemistry, University of Turku, Turku, Finland

^9^Accelerator Laboratory, Åbo Akademi University, Turku, Finland

**S1. Summary of pharmacokinetic variables after single oral doses of ORM-12741 in healthy males (n=18), for C_max_ and AUC_t_: geometric mean (CV%), for t_max_ and t_1/2_: mean (SD)**

| ORM-12741 dose (mg) | Number of subjects (N) | C_max_ (ng/mL) | t_max_ (h) | AUC_t_ (h×ng/mL) | t_½_ (h) |
| --- | --- | --- | --- | --- | --- |
| 0.3 | 2 | 1.21 (2) | 0.67 (0) | 4.50 (-)^2^ | 4.0 (0) |
| 1.0 | 2 | 2.69 (14) | 0.85 (0.3) | 8.45 (1) | 5.6 (0.1) |
| 10 | 5 | 62.6 (77) | 0.81 (0.2) | 129 (92) | 7.2 (3.3) |
| 30 | 5 | 121 (99) | 0.68 (0.02) | 316 (51) | 6.3 (1.3) |
| 60 | 4 | 174 (144) | 0.67 (0) | 732 (78) | 16.1 (9.5) |
| ^2^N = 1 |  |  |  |  |  |

**S2. Summary of the** **α_2C_-AR occupancy results by time point in the caudate nucleus and putamen**

| **Dose** | | **0.3 mg** | | | | | |
| --- | --- | --- | --- | --- | --- | --- | --- |
| Time | | 1 h (n = 1) | 3.5 h (n = 1) | | 6 h (n = 1) | | 12 h (n = 1) |
| Region | Caudate nucleus | -0.5% | -1.2% | | 5.4% | | -2.4% |
|  | Putamen | 1.5% | -0.3% | | 4.1% | | 0.2% |
|  |  |  |  | |  | |  |
| **Dose** | | **1 mg** | | | | | |
| Time | | 1 h (n = 1) | 3.5 h (n = 1) | | 6 h (n = 1) | | 12 h (n = 1) |
| Region | Caudate nucleus | 9.6% | 8.9% | | 11.2% | | 9.9% |
|  | Putamen | 5.2% | 5.7% | | 10.8% | | 8.8% |
|  |  |  |  | |  | |  |
| **Dose** | | **10 mg** | | | | | |
| Time | | 1 h (n = 3)^1^ | 3.5 h (n = 3) ^1^ | | 6 h (n = 2) ^1^ | | 12 h (n = 2) ^1^ |
| Region | Caudate nucleus | 32.2% | 28.1% | | 11.8% | | 9.9% |
|  | Putamen | 26.9% | 17.7% | | 5.6% | | 0.9% |
|  |  |  |  | |  | |  |
| **Dose** | | **30 mg** | | | | | |
| Time | | 1 h (n = 3) ^1^ | 3.5 h (n = 3) ^1^ | | 6 h (n = 2) ^1^ | | 12 h (n = 2) ^1^ |
| Region | Caudate nucleus | 64.6% | 17.3% | | 17.4% | | 0.7% |
|  | Putamen | 48.6% | 13.7% | | 15.2% | | 7.6% |
|  |  |  |  | |  | |  |
| **Dose** | | **60 mg** | | | | | |
| Time | | 1h (n = 4) ^1^ | | 3.5 h (n = 4) ^1^ | | 6.5 h (n = 4) ^1^ | |
| Region | Caudate nucleus | 64.8% | | 35.8% | | 18.7% | |
|  | Putamen | 49.1% | | 24.8% | | 13.0% | |
|  |  |  | |  | |  | |

^1^ Where there was more than 1 subject per ORM-12741 dose level and time point, mean values are shown

**S3. Individual α_2C_-AR occupancy results by time point in the caudate nucleus and putamen**

|  |  | Occupancy in the caudate nucleus (%) | | | |  | Occupancy in the putamen (%) | | | |  | Mean ORM-12741 concentration during PET measurement (ng/ml) | | | |
| --- | --- | --- | --- | --- | --- | --- | --- | --- | --- | --- | --- | --- | --- | --- | --- |
| Subject |  | 1 h | 3.5 h | 6-6.5 h | 12 h |  | 1 h | 3.5 h | 6-6.5 h | 12 h |  | 1 h | 3.5 h | 6-6.5 h | 12 h |
| 0.3 mg dose |  |  |  |  |  |  |  |  |  |  |  |  |  |  |  |
| 101 |  | -0.5 | -1.2 |  |  |  | 1.5 | -0.3 |  |  |  | 0.7 | 0.3 |  |  |
| 102 |  |  |  | 5.0 | -2.0 |  |  |  | 4.0 | 0.2 |  |  |  | 0.2 | 0.2 |
| 1 mg dose |  |  |  |  |  |  |  |  |  |  |  |  |  |  |  |
| 201 |  | 10 | 9.0 |  |  |  | 5.0 | 6.0 |  |  |  | 2.4 | 0.4 |  |  |
| 203 |  |  |  | 11 | 10 |  |  |  | 11 | 9.0 |  |  |  | 0.2 | 0.4 |
| 10 mg dose |  |  |  |  |  |  |  |  |  |  |  |  |  |  |  |
| 301 |  | 21 | 46 |  |  |  | 17 | 20 |  |  |  | 56 | 19 |  |  |
| 302 |  |  |  | 0.3 | 0.3 |  |  |  | 0.4 | -1.6 |  |  |  | 1.3 | 1.2 |
| 303 |  |  |  | 23 | 20 |  |  |  | 11 | 3.0 |  |  |  | 1.4 | 2.1 |
| 304 |  | 42 | 16 |  |  |  | 35 | 15 |  |  |  | 115 | 15 |  |  |
| 305 |  | 33 | 22 |  |  |  | 29 | 18 |  |  |  | 21 | 4.4 |  |  |
| 30 mg dose |  |  |  |  |  |  |  |  |  |  |  |  |  |  |  |
| 401 |  |  |  | 7.0 | -21 |  |  |  | 9.0 | 0.3 |  |  |  | 4.1 | 5.6 |
| 402 |  | 65 | 22 |  |  |  | 55 | 18 |  |  |  | 44 | 13 |  |  |
| 403 |  | 70 | 18 |  |  |  | 48 | 14 |  |  |  | 123 | 28 |  |  |
| 404 |  | 59 | 12 |  |  |  | 43 | 9.0 |  |  |  | 77 | 12 |  |  |
| 405 |  |  |  | 28 | 22 |  |  |  | 21 | 15 |  |  |  | 24 | 15 |
| 60 mg dose |  |  |  |  |  |  |  |  |  |  |  |  |  |  |  |
| 501 |  | 62 | 47 | 34 |  |  | 48 | 32 | 24 |  |  | 254 | 88 | 67 |  |
| 502 |  | 56 | 18 | -12 |  |  | 46 | 14 | -7.0 |  |  | 51 | 13 | 8 |  |
| 503 |  | 70 | 45 | 20 |  |  | 49 | 30 | 12 |  |  | 45 | 54 | 16 |  |
| 504 |  | 71 | 34 | 33 |  |  | 53 | 24 | 22 |  |  | 114 | 39 | 29 |  |

**S4. AEs after start of ORM-12741 treatment (subject count, %)**

| Preferred term | 0.3 mg N = 2 | 1 mg N = 3 | 10 mg N = 5 | 30 mg N = 5 | 60 mg N = 4 |
| --- | --- | --- | --- | --- | --- |
| Feeling hot |  |  |  | 1 (20%) |  |
| Sinusitis |  | 1 (33.3%) |  |  |  |
| Gamma-glutamyltransferase increased |  |  |  |  | 1 (25%) |
| Headache | 2 (100%) |  | 2 (40%) | 1 (20%) | 1 (25%) |
| Cough |  | 1 (33.3%) |  |  |  |
